# The association between methamphetamine use and number of sexual partners in men who have sex with men: a systematic review and meta-analysis

**DOI:** 10.1186/s13011-022-00453-7

**Published:** 2022-04-09

**Authors:** Salah Moradi, Yousef Moradi, Khaled Rahmani, Bijan Nouri, Ghobad Moradi

**Affiliations:** 1 Department of Epidemiology and Biostatistics, Faculty of Medicine, Kurdistan University of Medical Sciences, Sanandaj, Iran; 2 Social Determinants of Health Research Center, Research Institute for Health Development, Kurdistan University of Medical Sciences, Sanandaj, Iran

**Keywords:** Methamphetamine, Men Who Have Sex with Men, Sexual Partners, Meta-Analysis

## Abstract

**Background:**

Methamphetamine use in men who have sex with men population is significantly higher than that in the general population. Meth use can cause high-risk sexual behaviors, such as having sex with a variety of sexual partners. The aim of this study was to determine the association between meth use and the number of sexual partners in MSM.

**Methods:**

Searching international databases (PubMed (Medline), Scopus, Web of Sciences, Embase (Elsevier), PsycInfo (Ovid), Cochrane CENTRAL (Ovid)) until March 2021 was performed in this meta-analysis using appropriate keywords terms to identify related articles. After retrieving articles in these databases, screening was performed based on the title, abstract and full text of the articles, and the final related studies were selected and evaluated using the Newcastle Ottawa scale checklist.

**Results:**

The sample size consisted 18,455 people in this study, including four cohort studies with a sample size of 15,026 MSM and four case–control studies with a sample size of 3429 MSM. The results of meta-analysis showed that meth use increased the number of sexual partners in MSM (RR: 3.70; % 95 CI: 2.04—6.70). The results of subgroup analyze based on the number of sexual partners showed that in MSM taking meth, the risks of having one to three, four to five, and six or more than six sexual partners were respectively 2.82, 2.98 and 5.89 times higher than those in MSM who did not take meth.

**Conclusion:**

The results showed that meth uses in MSM increased the number of their sexual partners. Due to the fact that increasing the number of sexual partners and high-risk sexual behaviors increase the risk of contracting sexually transmitted diseases such as HIV, it is necessary to adopt control programs to prevent meth use by this group, or to implement programs of reduction in the risk of STIs for this group.

## Background

Methamphetamine (meth) is currently a global public health concern and is estimated to have been used by 14 to 53 million people worldwide in 2015 [1]. Meth is an addictive substance used in many different ways. It is usually synthesized illegally in laboratories using over-the-counter drugs, including ephedrine or adrenaline [2, 3]. Meth increases alertness, energy, and self-esteem. It causes euphoria with more energy and less fatigue, which is called "rash" or "flash" usually accompanied by pleasure, movement and increased concentration [2–4]. Short-term effects of meth include increased alertness, decreased appetite, headache, dizziness, fever, increased heart rate, increased blood pressure, and increased respiratory rate [2, 3, 5]. By regularly taking meth for a long period of time, the risk of developing psychosis or psychological symptoms increases [6]. These symptoms include violent behaviors, paranoia, and delusions which pose risks to users and pose challenges for medical and health care professionals. Globally, North America with 3.92 million users ranks first in the world in terms of prevalence of meth users, and East Asia is in the second place [7, 8]. The use of meth and amphetamine stimulants has increased in the United States over the past 20 years [9, 10]. The prevalence of meth use in Canada is about 0.2% of the total population [11]. The number of meth-related accidents increased by 590% between 2010 and 2017 [11]. The united nations office on drugs and crime (UNODC) reported that 547.7 kg of meth were seized in Canada in 2016, which had increased up about 330% from the previous year [11]. According to a survey conducted by the British Columbia Centre for Disease Control (BC Center), which estimated the trend in substance use among clients for harm reduction across the state (812 sites), found that meth use has increased from 16.6% in 2012 to 47% in 2015 over the past seven days among respondents [12, 13]. The meth groups is a common substance use in populations at risk for HIV, especially sexual minorities [14]. Meth use is highly prevalent among MSM and bisexuals [15, 16]. A small number of studies which had examined the association between meth use and heterosexual behaviors showed that meth use was associated with increased libido, increased sexual activities, prolonged sexual activities, increased sexual confidence, more sexual partners, casual sexual partners, anal intercourse, and sex trafficking with drugs or money. This is why some people use these substances to increase sexual desire and prolong sexual intercourse. Use of these substances increases high-risk sexual behaviors [17]. Behaviors which may be considered high-risk sexual ones include unprotected intercourse, intercourse under the influence of drugs, intercourse with injecting drug users, having multiple sexual partners, the early age of onset of sexual intercourse, and intercourse with female sex workers in exchange for money. Adverse effects of high-risk sexual behaviors include sexually transmitted infections (STIs), human immunodeficiency virus (HIV), unwanted pregnancies, premature pregnancies, and abortion. The literature shows that all the effects of high-risk sexual abuse are more prevalent in patients who use drugs than in the general population [18]. The prevalence of lifetime substance use among MSM is significantly higher than that among heterosexual men or women [19]. This substance is commonly used in sexual settings, including sex clubs, major gay dating sites such as bars, dance clubs, and school parties, and environments which facilitate the risk of increase in unprotected sexual behaviors [20]. Heterosexuals also appear to increase the risk of contracting STIs, especially when both partners use it [6]. Potential consequences of meth use are transmission of HIV and other STIs [17]. There is a strong association between meth use and high-risk sexual behaviors related to HIV, observed in studies on MSM, which is consistent with increase in prevalence of HIV and syphilis in MSM who use these substances compared to MSM who do not use these substances [21]. The results of related studies conducted in around the world also emphasize this. For example, The McKetin. R et al. show that meth use was increased the risk of having unsafe sex, especially sex with multiple partners [22]. Given the above and the importance of the association between meth use and having several sexual partners in MSM and subsequently increasing the risk of STIs, the aim of this study was to determine the association between meth use and the number of sexual partners in MSM using meta-analysis method.

## Methods

This systematic review and meta-analysis was based on preferential reporting items for systematic review and meta-analysis (PRISMA) according to the following 5 steps of search terms and search strategy, screening and selection, data extraction, risk of bias, and meta-analysis [23].

### Search strategy and information sources

In the present study, a comprehensive search of articles was conducted in the international databases of PubMed (Medline), Scopus, Web of Sciences, Embase (Elsevier), PsycInfo (Ovid), Cochrane Central (Ovid) from January 1990 to March 2021. The main keywords for searching in the present meta-analysis were meth, multiple sex partners, and MSM. Synonyms of these keywords were extracted from Mesh and Emtree to formulate search and search strategy in different databases. After searching in the mentioned databases, manual search was performed by checking the references of selected related articles.

At the end of the search strategy, and checking for duplicates of articles, the studies were entered into EndNote software (version 8) for screening based on titles, abstracts and full texts, respectively. All screening cases were performed according to the study inclusion criteria. It should be noted that the strategy of searching and screening articles was independently done by the two authors (SM and YM) and the disputes were resolved by a third expert (GM).

### Eligibility criteria

Preliminary studies in which the study population was MSM, meth use was considered as exposure, and the number of sexual partners was considered as the outcome, were included in the present meta-analysis. Also, in terms of the study type, only cohort or case–control studies were considered because the aim of this meta-analysis was to determine the association between meth use and the number of sexual partners of MSM. In addition, the indicators of measuring the desired association for meta-analysis were the odds ratio (OR), risk ratio (RR), and hazard ratio (HR) in selected studies.

### Exclusion criteria

In the present meta-analysis, the letter to the editor, review studies, meta-analyses, case reports or case series with non-MSM populations, clinical or interventional trials, studies with measurement indicators other than OR, RR, or HR were excluded from analysis.

### Data extraction and analysis

The data from each eligible study were extracted by the two independent researchers (SM and YM) and recorded in Excel while differences were discussed in the presence of a third expert (GM) to reach an agreement. Each OR, HR or RR was reported and the corresponding 95% confidence interval (CI) was extracted from each study for determining the association between meth use and the number of sexual partners of MSM. In addition to the effect size (ES), information such as name of the first author, year of publication, country, profile of the participant population, age range or mean age, gender, number of participants and cases, time period of follow-up for prospective studies, and number of sexual partners was extracted. If a study reported its findings based on the number of sexual partners, that study was separately listed several times based on the different categories of sexual partners in the data extraction table.

### Quality assessment

Quality of studies available in the current meta-analysis was evaluated using the Newcastle Ottawa Scale (NOS) [24]. Based on this scale, according to the following parameters, a maximum of 9 points was awarded to each study, which included four points for the selection of participants, two points for the comparison, and 3 points for the evaluation of the results. A study with a score of 7–9, was considered with high quality (in terms of the occurrence of bias, in the low category), with a score of 4–6, with medium quality (in terms of the occurrence of bias, in the moderate category) and with a score of 0–3, with low quality (in terms of the occurrence of bias, in the high category).

### Statistical analysis

All analyzes were performed using STATA software, version 16 [25]. First, logarithm and standard deviation of OR, RR and HR logarithm and CI 95% were calculated for meta-analysis. The effect size of the reported studies was different so that in the case–control studies, the OR was reported and in the cohort ones, the RR or HR was reported. So, first, a general analysis of all studies was performed by reporting the relative risk index [26]. The random effect model was used for the analysis. To check the heterogeneity in the meta-analysis, I square and Q Cochrane indicators were applied. According to Cochrane criteria, if I square percentage is 0 to 25%, there will be no heterogeneity, 25 to 50%, heterogeneity will be low, 50 to 75%, heterogeneity will be high but acceptable, and finally 75 to 100%, heterogeneity will be very high. To identify the main source of heterogeneity, subgroup analysis based on important variables (the number of sexual partners, number of samples, type of studies and geographical location) and meta-regression analysis were used. The publication bias was examined using the Egger regression asymmetry test [27]. Sensitivity analysis was also performed using a random effect model in which each study was excluded from the research to evaluate the effect of that study on the overall estimate.

## Results

### Qualitative results

**Fig. 1:**
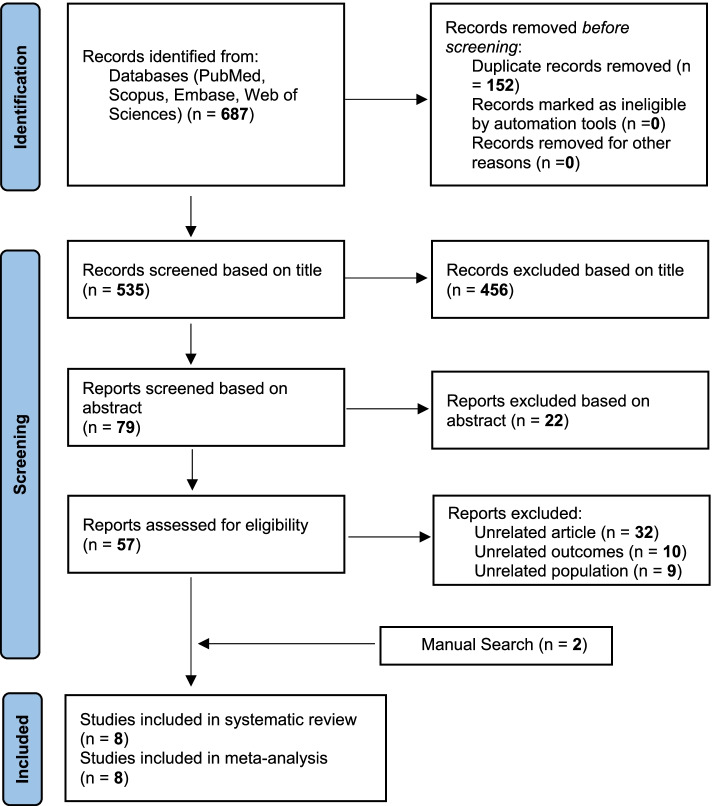
PRISMA 2020 flow diagram for new systematic reviews which included searches of databases and registers only

After searching international databases and screening articles by title, abstract and full text, 10 studies related to the topic and aim of the meta-analysis remained. After a more detailed review of the article full text, 4 more studies were removed because in their methods, a combination of drug use, different from the aim of this study, which was meth use, was examined as exposure. Finally, after performing a manual search, two articles done by Hoenigl and Prestage were added to the number of final studies [28, 29]. At the end, 8 articles including 4 articles from cohort studies (Piyaraj et al. [30], Plankey et al. [31], Prestage et al. [29] and Hoenigl et al. [28]) and 4 articles from case–control studies (Hirshfield et al. [32], Drumright et al. [33], Brown et al. [34] and Fernandez et al. [35]) were selected for meta-analysis. Among them, the main outcome of Piyaraj study was to determine, and classify the number of sexual partners into two groups of one to five as well as 6 and above, Plankey study into three groups of one, two to four and more than five sexual partners, Hirshfield study into two groups of two to five and more than six partners, Drumright study, three partners, Brown and Fernandez study, more than one partner, and finally, Hoenigl and Prestage studies, more than four sexual partners. The flow chart of the study selection was shown in (Fig. 1).

The study characteristics were presented in Table 1. The total number of participants in these studies ranged from 75 to 8905 people with an age range of 18 to 60 years and older. In total, 18,455 participants (cohort studies = 15,026 and case–control studies = 3429) were included in 8 articles studied by the current meta-analysis. The period time of follow-up varied between 12 and 80 months. All studies were performed on a MSM population. Of the eight articles, six were conducted in the United States and two in Australia and Thailand. Meth use was assessed in 7 studies by interviews and self-reports, and in Hoenigl article, it was detected by rapid testing. Among the case–control studies, the highest and lowest sample sizes were related to the studies of Hirshfield et al. [32] (*n* = 2643) and Brown et al. [34] (*n* = 75), respectively. Among prospective studies, the highest and lowest sample sizes were related to the studies of Hoenigl et al. [28] (*n* = 8905) and Prestage et al. [29] (*n* = 764), respectively. The quality assessment scores of the articles ranged from 7 to 9 based on the NOS checklist. In this research, five studies received a score of seven, and two studies received a score of eight and one study received a score of nine (Table 2).

**Table 1:** The characteristics of Studies that evaluation the relationship between methamphetamine uses and multiple sexual partners in MSM

Authors (Years)	Country	Type of Study (Cohort or Case Control)	Study Population	Duration of follow-up (month)	Sampling	Exposure assessment	Age (Median)		Total number of male partners		Effect size (OR with % 95 CI) or (RR with % 95 CI)	Outcomes
Piyaraj et al. (2018) [19]	Thailand	Cohort	1372 MSM	60	Volunteered ^Self−selected men^	Self-reported	Total:26		1–5	786 (57%)	HR: 2.7 (95% CI: 0.6–11)	Increase
							18–21	264 (19%)				
							22–29	724 (53%)	≥ 6	522 (38%)	HR: 9.7 (95% CI: 2.4–39.7)	Increase
							≥ 30	384 (28%)				
Planky et al. (2007) [20]	USA	Cohort	4003 MSM	84	Volunteered ^Self−selected men^	Self-reported	Total:33.4		1		HR: 2.71 (95% CI: 1.81–4.04)	Increase
							18–25	672 (17%)				
							26–35	1795 (45%)	2–4		HR: 7.79 (95% CI: 5.17–11.74)	Increase
							36–45	1148 (28%)				
							46–55	323 (8%)	≥ 5		HR: 13.57(95% CI: 8.43–21.84)	Increase
							56 +	65 (2%)				
Hirshfield et al. (2004) [21]	USA	A Nested Case–Control	2643 MSM	-	Volunteered ^Self−selected men^	Self-reported	Total: 33		2–5	1103(%44)	OR: 1.6 (95% CI: 0.6 -3. 9)	Increase
							18–29	1218(46.08)				
							30–39	728(27.54)	≥ 6	888 (%36)	OR: 3.3 (95% CI: 1.4- 7.8)	Increase
							40 +	697(26.37)				
Drumright et al. (2009) [22]	USA	Case–Control	145 MSM	-	Volunteered ^Self−selected men^	Self-reported	Total:32.4		3		OR: 2.77 (95% CI: 1.2–6.6)	Increase
Brown et al. (2017) [23]	USA	Case–Control	75 MSM	-	Volunteered ^Self−selected men^	Self-reported	Total: 42.8		1		OR: 5.7 (95% CI: 1.7– 19.3)	Increase
							Total: 40.7		> 1		OR: 10.5 (95% CI: 2.2– 50.6)	Increase
Hoenigl et al. (2015) [17]	USA	Cohort	6676 MSM	12	Random	Rapid Test	Total: 32		> 5		OR: 3.19 (95% CI: 2.52– 4.02)	Increase
Prestage et al. (2009) [18]	Australia	Cohort	746 MSM	12	Volunteered	Self-reported	Total: 39.8		> 4		OR: 1.98 (95% CI: 1.23- 3.19)	Increase
Fernandez et al. (2007) [24]	USA	Case–Control	566 MSM	-	Random	Self-reported	Total: 30.86		> 1		OR: 1.038 (95% CI 1.017–1.06)	Increase

*Abbreviation*: *MSM* Men sex with men, *OR* Odds ratio, *RR* Relative risk, *HR* Hazard ratio

**Table 2:** Methodological Quality scores included cohort studies using Newcastle–Ottawa scale

Study	Selection			Comparability	Outcome/Exposure		Study score/9
	Representativeness of the sample	Non-respondents	Ascertainment of the exposure (risk factor)	The subjects in different outcome groups are comparable, based on the study design or analysis. Confounding factors are controlled	Assessment of the outcome/Exposure	Statistical test	
**Hirshfield et al. (2004) **[21]	*	*	*	**	**	-	7
**Fernandez et al. (2007) **[24]	*	*	*	**	**	-	7
**Drumright et al. (2009) **[22]	*	*	*	**	**	*	8
**Brown et al. (2017) **[23]	*	*	**	*	*	*	7
**Planky et al. (2007) **[20]	*	*	*	**	*	*	7
**Prestage et al. (2009) **[18]	*	*	*	**	*	*	7
**Hoenigl et al. (2015) **[17]	*	*	*	**	**	*	8
**Piyaraj et al. (2018) **[19]	**	*	*	**	**	*	9

### Quantitative results

**Fig. 2:**
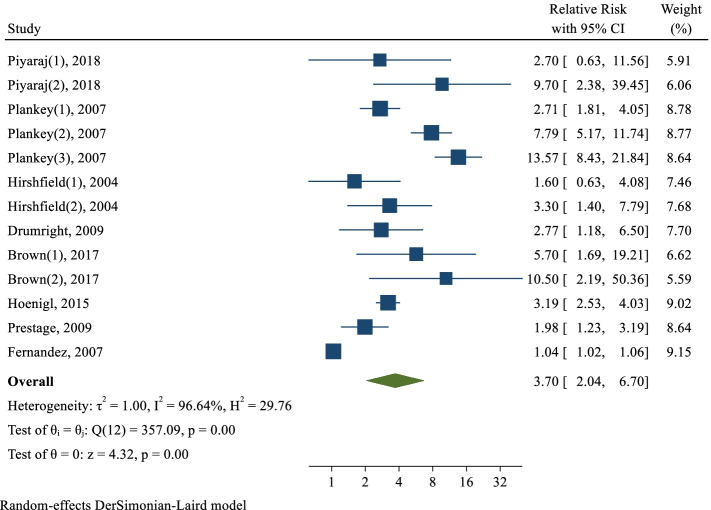
The pooled relative risk of methamphetamine on the risk of multi-partner sex

**Fig. 3:**
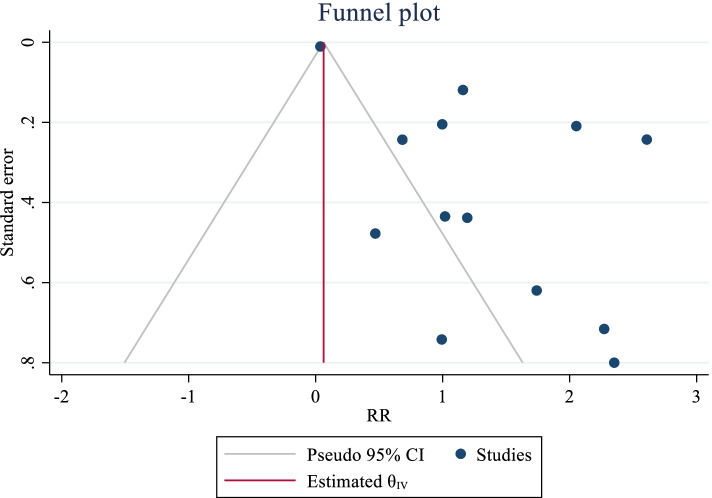
The publication bias of the association between methamphetamine and the risk of multi-partner sex

The meta-analysis results on the association between meth use and the number of sexual partners of MSM showed that eight studies with a total of 18,455 participants were included in the meta-analysis. After combining the results of these articles, regardless of the study type, meth uses in MSM increased the risk of having multiple sexual partners by 3.70 times compared to MSM who did not use meth (RR: 3.70; 95% CI: 2.04—6.70; I2: 96.64%; P: 0.001) (Fig. 2). The results of publication bias using the eggers test showed no bias (B: 1.22; SE: 0.98; P: 0.214) (Fig. 3).

#### Subgroup analysis

In the present meta-analysis, in order to find the main sources of heterogeneity in determining the overall effect of meth use on the presence of several sexual partners, a subgroup analysis was performed based on the variables of number of sexual partners, number of samples, type of studies and geographical location.

#### Subgroup analysis based on the number of sexual partners

The results of the analysis of subgroups based on the number of sexual partners showed that meth use increased the risk of having one to three sexual partners by 2.82 times (RR: 2.82; 95% CI: 1.30—6.11; I2: 90.66%; *P* < 0.001). Also, meth use increased the risk of having 6 or more sexual partners by 5.89 times (RR: 5.89; 95% CI: 2.37—14.65; I2: 90.10%; *P* < 0.001), respectively (Table 3).

**Table 3:** Subgroup analysis based on fixed-effects models for the association between methamphetamine and multiple sex partner

		**Number of effect sizes**	**(I**^**2**^**)**^**1**^	**P**^**2**^	**RR (95%CI)**^**3**^	**P**_**-be**_^**4**^
**Methamphetamine based on multiple sex partner**						
**Overall**		13	96.64	< 0.001	3.70 (2.04–6.70)	
**Number of Sexual Partners**	1 to 3	5	90.66	< 0.001	2.82 (1.30—6.11)	< 0.001
	4 to 5	4	86.63	< 0.001	2.98 (1.19 -7.47)	
	6 or more	4	90.10	< 0.001	5.89 (2.37–14.65)	
**Country**	USA	10	97.35	< 0.001	3.77 (1.89 -7.53)	< 0.001
	Non-US countries	3	55.09	0.110	3.17 (1.26—8.00)	
**Sample size**	Under 1000 Sample	5	85.72	< 0.001	2.52 (1.25–5.09)	< 0.001
	1000 Sample or above	8	85.43	< 0.001	4.44 (2.66–7.40)	
**Age**	26–33	7	94.64	< 0.001	2.55 (1.31–4.93)	< 0.001
	Greater than 33	6	89.60	< 0.001	5.38 (2.65–10.92)	

*Abbreviation*: *RR* Relative risk

^1^Inconsistency, percentage of variation across studies due to heterogeneity

^2^Obtained from the Q-test

^3^Obtained from the fixed-effects model

^4^Heterogeneity between groups

#### Subgroup analysis based on the geographical location

In this meta-analysis, the geographical location was divided into two categories of studies performed in American countries and in non-American countries due to the small number of studies in other countries. The results of the meta-analysis based on the geographical location showed that American MSM were more at risk of increasing the number of sexual partners after taking meth than non-American MSM (RR: 3.77; 95% CI: 1.89—7.53; I2: 97.35%; *P* < 0.001 vs. RR: 3.17; 95% CI: 1.26—8.00; I2: 55.09%; P: 0.110) (Table 3).

#### Subgroup analysis based on the number of samples participating in the study

In this meta-analysis, five studies had a sample size of less than 1000 MSM, and 8 studies had a sample size equal to or greater than 1000 people. After meta-analysis and combining the results of articles with a sample size of less than 1000 people, the effect of meth uses on increasing the number of sexual partners was 2.52 (RR: 2.52; 95% CI: 1.25—5.09; I2: 85.72%; *P* < 0.001) while for studies with a sample size equal to or more than 1000 people, this effect was equal to 4.44 (RR: 4.44; 95% CI: 2.66—7.40; I2: 85.43%; *P* < 0.001) (Table 3).

#### Subgroup analysis based on the age of study participants

The age of MSM studied in the initial articles was divided into two group of 26 to 33 years and 33 years and above. Meta-analysis was performed to determine, and compare the meth use and the increase in sexual partners of these two age groups. The results showed that in the age group of 26 to 33 years of MSM, meth use increased the risk of increasing the number of sexual partners by 2.55 times (RR: 2.55; 95% CI: 1.31—4.93; I2: 94.64%; *P* < 0.001) while it increased this risk by 5.38 times in the age group of participants over 33 years (RR: 5.38; 95% CI: 2.65—10.92; I2: 89.60%; *P* < 0.001) (Table 3).

## Discussion

The aim of the present meta-analysis was to determine the association between meth use and an increase in the number of sexual partners in MSM. The results of meta-analysis showed that meth use significantly increased the number and variety of sexual partners so that MSM who took meth were 3.70 times more likely to have sex with multiple sexual partners simultaneously or at different times than MSM who did not use meth. The results of some other studies were also consistent with those of the present meta-analysis [31, 32]. The reason for this association is that meth use increases sexual desire, sexual activities, and sexual confidence in MSM, as well as it prolongs sexual activities. Following these consequences, meth use eventually increases high-risk sexual behaviors such as having multiple sexual partners, the tendency to have anal intercourse, and the sex trade for drugs or money, among which having multiple sexual partners is very important due to the development of STIs [17]. In a cohort study of 4,003 samples, conducted by Plankey et al. in 2007, the results showed that meth use increased the risk of having a sexual partner by 2.71 times while the risk of having two to four or more sexual partners increased by 7.79 times. This study also showed that the risk of increasing the number of sexual partners, higher than five people after taking meth was equal to 13 times [31]. In line with the results of the present meta-analysis, another study performed by Hirshfield et al. in 2004 on 2,643 participants showed that meth use increased the risk of having two to five or more sexual partners [32]. In a cross-sectional study conducted by Taylor et al. in 2007 on 2915 individuals, the results showed that the prevalence ratio of meth use with multiple sexual partners was 2.2, and this result was related to the prevalence of meth use and multiple sexual partners [36]. Another cross-sectional study done by Garofalo et al. in 2007 on 310 samples showed that the prevalence of meth use among MSM with multiple sexual partners was 4.6 [37]. These results showed that the prevalence of meth use was related to having multiple sexual partners, which were in line with the results of the meta-analysis. Meth use leads MSM to increase the number of sexual relations with different people and to have anal intercourse [38]. In some European countries, the use of drugs and psychotropic substances such as meth during sexual intercourse is common among many lesbians, gay, bisexual, transgenders (LGBT), and MSM. This is because taking meth causes more mania or happiness than other drugs such as cocaine, etc. [39, 40]. By experiencing these substances, people will become more sexually active. Increased libido increases the risk of high-risk sexual behaviors, and increased high-risk behaviors can also increase the risk of transmitting diseases such as HIV and hepatitis.

The result of and the association between meth use and the number of sexual partners can be very significant. However, the degree of heterogeneity in the final findings should be considered, which indicates a significant difference in the methodology of the initial studies included in the meta-analysis. In the present study, heterogeneity was investigated using subgroup analysis and as it is clear from the results of subgroup analysis, the percentage of heterogeneity decreased in some subgroups. For example, when combining the results of not American studies and preliminary ones which had examined meth use with one sexual partner, the percentage of heterogeneity decreased, and it can be concluded that in these subgroups, the studies were homogeneous. Significant differences in the statistical population and the number of sexual partners studied in the primary articles can be considered as a source of heterogeneity in the overall association between meth use and the number of sexual partners. On the other hand, meth is more common in the United States than in other countries. Also, most meth users in the United States are in the age group of 18 to 25 years, and most are men. Therefore, the occurrence of unprotected sex, and having several sexual partners are highly expected in this age group in the United States after taking meth. These scientific justifications are in line with the results of the meta-analysis presented in Table 3, which have shown that meth use increases the number of sexual partners of American MSM more than MSM of other countries [41]. In addition, the results of subgroup analyze in this meta-analysis showed that the association between meth use and increasing the number of sexual partners in MSM aged 26 to 33 years was less than that of the age group of 33 years and above. The reason for this can go back to various topics and aspects. People lose more control at older ages due to meth use and are more likely to have sex with several people simultaneously or at different times. On the other hand, it is possible that this age group has more experience in meth use than the age group of 26 to 33 years, which leads to a greater impact of these substances on this age group. This is because meth use in the early years of consumption has a greater effect on causing hallucinations and cognitive behaviors, and it is possible that studied MSM aged 26 to 33 years have been in the first stage after taking meth. Other reasons for this include that taking meth leads a MSM to engage in oral, vaginal, anal, violent, or unprotected sex which may become normal for people over the age of 33 years, but for the age group of 26 to 33 years, there are still some limitations or fears in this regard, which are the reason for the lower effect of meth on the increase in the number of sexual partners in the age group of 26 to 33 years than that in the age group of 33 years and above [42–44].

In 2017, 28.9 million meth users were estimated, who were 0.6 percent of the global population aged 15–64. The highest prevalence among the population aged 15 to 64 years was in North America (2.1%) and Oceania (1.3%). The form of used amphetamines considerably varies from region to region. In North America, medicines containing stimulants are mostly used for non-medical purposes. Crystalline meth is used in East and Southeast Asia and Oceania (Australia), and amphetamine is used in Western and Central Europe and the Middle East. Since 2010, the use of amphetamines has relatively been stable in most Western and Central European countries. But in North America, there are signs of increased meth use while meth use, especially crystalline meth, has been reported in East and Southeast Asia [45]. Regarding MSM, if they take meth, the tendency to take different sexual Viagra and different examples of sexual enhancers will significantly increase. This will greatly increase the number of sexual partners. As a result, sexual desires for unprotected sex, and violent sexual behaviors will occur, leading to STIs such as HIV. The results of previous studies have shown that meth use in the MSM leads to increases the risk of HIV and STIs, and it can reduce adherence to treatment and increase drug or multi-drug resistance in HIV-infected MSMs [46–49]. Due to the characteristics and behaviors that will be created after using meth, if using this substance in HIV-infected MSMs, high-risk sexual behaviors such as having unsafe sex, having sex with several people in exchange for money, having different sexual partners, and consuming alcohol while having sex will increase [50, 51]. Other public health problems associated with meth use in HIV-infected MSMs include the potential to transmit new resistant strains to other people, impaired immunity after receiving medical care or drug treatment, and an increase in high-risk sexual behaviors, especially the number of sexual partners, and ultimately the increasing complexity of the HIV epidemic in the world [52, 53]. It should be noted, however, that it is not yet clear exactly how much meth use increases the progression and transmission of HIV, or the development of new refractory strains, and further studies are needed. This study was the first meta-analysis in the world to determine the association between meth use and the increase in the number of sexual partners in MSM, the results of which showed a significant increase in the number of sexual partners following the use of meth. One of the limitations of this study was the small number of cohort and case–control studies published about determining the association between meth use and an increase in the number of sexual partners. Also, the lack of analysis of different subgroups based on important variables such as HIV status due to the lack of reporting these variables in the initial studies, was another limitation of this research. This prevented the identification of the main sources of heterogeneity in this meta-analysis. Because of the small number of articles, to find a stronger and more significant association with considering HIV status, the need is felt for performing studies with a larger sample size in the form of cohort research.

## Conclusion

The results of the present meta-analysis showed that meth use in MSM increased the number of sexual partners and provided evidence to strengthen this association. Due to the association between increasing the number of sexual partners, high-risk sexual behaviors and the incidence of STIs such as HIV, HBV and HCV, it is necessary to adopt control programs to prevent the use, and reduce the risk of STIs in this group, especially among meth users. Implementing programs of harm reduction for MSM meth users, including provision of syringes, use of methadone, and provision of condoms, is very effective in controlling high-risk sexual behaviors and reducing the incidence of STIs in meth users.

## Data Availability

Data and materials are available within the supplementary materials and further information can be made available by request to corresponding author.

## Abbreviations

MSM: Male who have sex with Men
Meth: Methamphetamine
HIV: Human Immunodeficiency Virus
STIs: Sexually Transmitted Infections
HCV: Hepatitis C Virus
HBV: Hepatitis B Virus
LGBT: Lesbians, Gay, Bisexual, Transgenders
NOS: Newcastle Ottawa Scale
OR: Odds Ratio
RR: Risk Ratio/Relative Risk
CI: Confidence Interval
PRISMA: Preferred Reporting Items for Systematic Reviews and Meta-Analyses
I2: I Square
